# Diabetes-related foot disease research in Aotearoa New Zealand: a bibliometric analysis (1970–2020)

**DOI:** 10.1186/s13047-022-00528-5

**Published:** 2022-03-21

**Authors:** Matthew Carroll, Ibrahim Saleh Al-Busaidi, Kirsten J. Coppell, Michele Garrett, Belinda Ihaka, Claire O’Shea, Justina Wu, Steve York

**Affiliations:** 1 Department of Podiatry, School of Clinical Sciences, Faculty of Health & Environmental Sciences, Auckland University of Technology, Auckland, New Zealand; 2 Department of General Practice, University of Otago Christchurch, Christchurch, New Zealand; 3 Department of Medicine, Dunedin School of Medicine, University of Otago, Dunedin, New Zealand; 4 Community and Long Term Conditions Directorate, Auckland District Health Board, Auckland, New Zealand; 5 Waikato District Health Board, Hamilton, New Zealand; 6 High Risk Foot Clinic, Northland District Health Board, Whangarei, New Zealand

**Keywords:** Bibliometric analysis, Diabetes-related foot disease, Diabetes mellitus, Diabetic foot, Research, Aotearoa New Zealand

## Abstract

**Background:**

The aim of this bibliometric study was to examine trends in the quality and quantity of published diabetes-related foot disease (DRFD) research in Aotearoa/New Zealand (NZ) over the past five decades.

**Method:**

In July 2021, the Scopus® database was searched for DRFD-related publications (1970–2020) using predetermined search and inclusion criteria. Bibliometric data were extracted from Scopus® and Journal Citation Reports. Retrieved bibliometric indicators were analysed in Biblioshiny, an R Statistical Software interface and reported using descriptive statistics.

**Results:**

Forty-seven DRFD-related articles were identified. The annual number of publications showed a significant upward trend increasing from one in 1988 to a peak of six in 2018 (*P* < 0.001). The majority of identified articles (*n* = 31, 66%) were published in the last decade (2011–2020). Basic/clinical research accounted for 87% (*n* = 41) of publications and 14 (30%) investigated the screening and/or prevention of DRFD. The average citation per article was 20.23 (range: 0–209) and the median impact factor was 4.31 (range, 1.82–79.32). Over a third of articles (36%) had an international authorship network. Funding was reported in 15 (32%) articles; 12 (26%) were supported by public national grants vs. three (6%) reporting industry-sponsorship.

**Conclusion:**

DRFD articles authored by NZ researchers have increased over the past five decades. Despite NZ researchers having increased their global impact through collaborative networks, most of the research was classified as low-level evidence, with limited focus on Indigenous Māori and limited financial support and funding. Increased funding for interventional research is required to enable a higher level of evidence-based and practice-changing research to occur. With rates of diabetes-related amputations higher in Māori future research must focus on reducing inequalities in diabetes-related outcomes for Māori by specifically targeting the prevention and screening of DRFD in primary care settings in NZ.

**Supplementary Information:**

The online version contains supplementary material available at 10.1186/s13047-022-00528-5.

## Background

Diabetes-related foot disease (DRFD) is one of the most devastating, but potentially avoidable complications of diabetes [1, 2]. DRFD is defined as a foot affected by infection, ulceration or destruction of tissues of the foot of a person with currently or previously diagnosed diabetes mellitus, usually accompanied by neuropathy and/or PAD in the lower extremity [3]. Diabetes-related foot ulcers are the most frequently recognised complication of DRFD and a major risk factor for, and nearly always precede, diabetes-related lower-extremity amputation (DRLEA) [4–6]. DRLEA is one of the most substantial and debilitating consequences of diabetes [7].

Diabetes is common worldwide, affecting an estimated 10.5% of adults 20–79 years globally in 2021 with prevalence rates varying between countries [8]. In Aotearoa/New Zealand (NZ), in 2008/09, the prevalence of diabetes (diagnosed and undiagnosed) among those aged ≥15 years was 7.0% overall, and ethnic specific rates were 9.8, 15.4 and 6.1% among Māori, Pacific peoples and non-Māori non-Pacific peoples, respectively [9]. The age standardised rate for major DRLEAs in NZ is 6.4 per 100,000, which is almost double the rate for Australia and the United Kingdom [10, 11]. More than half (58%) of DRLEAs are attributable to diabetes [12] and Indigenous Māori are more likely to experience both major and minor DRLEA than their NZ European counterparts, with males bearing a higher burden [13–15]. Gurney et al. demonstrated that Māori with diabetes were 65% more likely to undergo major DRLEA than NZ European/Other people with diabetes [12]. Internationally indigenous populations have poorer diabetes foot outcomes and higher rates of risk factors, which occur at a younger age compared with non-indigenous populations [16]. Understanding DRFD research quantity and quality in the NZ context is important, as Indigenous Māori people not only have high rates of DRLEA [13, 17] but also fare worse in many other diabetes-related health measures [15, 17].

Whilst the understanding of DRFD has been advanced over many decades based on international research, the contribution of locally NZ driven DRFD-related research appears to be limited [13, 15, 17]. Consequently, we do not know if NZ based DRFD research is targeting the areas specific to achieving a reduction in DRLEA and improved health outcomes. The objective of this study was to provide the first comprehensive bibliometric analysis of DRFD research generated by NZ based researchers in order to present a “big picture” of extant research. Specifically, the study aimed to identify underlying patterns in DRFD publications, author-specific contributions, the volume of scholarly work over time, the degree of national and international collaborations, and the major topics/areas of research focus.

## Methods

### Data source

This bibliometric analysis of NZ DRFD publications between 1970 and June 2021 was conducted in July 2021 using data sourced from the Scopus® database (Elsevier, Amsterdam, Netherlands). The Scopus® database was selected as it enables search by document, author or affiliation, or use, with the ability to refine results by author and publication characteristics. It has the largest abstract and citation database of research literature [18]. As of January 2020, Scopus® had in excess of 25,100 active titles and over 550 articles in press [19]. Additionally, Scopus® includes a more expanded spectrum of journals than PubMed and Web of Science®, and its citation analysis is faster and includes more articles than the citation analysis of Web of Science® [20].

### Search strategy

The search strategy was developed through a staged process, involving adaptation of a search strategy used in a previous bibliometric analysis of DRFD conducted by some authors of this study [21]. Initially, Al-Busaidi et al.’s search strategy was run in Scopus® [21], with the 15 most cited articles retrieved. Author keywords, Medical Subject Headings (MeSH) classifications, and Emtree (Embase subject headings) terms were then downloaded from Scopus® and exported into NVivo Qualitative Data Analysis Software (QSR International Pty Ltd. Version 12, 2018) and analysed by text query analysis to obtain word frequency counts. Keywords were reviewed and discussed by the authors to develop the final search strategy displayed in Table 1.

**Table 1:** Scopus® search strategy (1970–2020)

Keywords	
1. Diabet*	
2. Neuropathy	
3. Arterial	
4. Amputation	
5. Infect*	
6. Ulcer	
7. Wound	
8. Foot or feet	
SEARCH STRATEGY	
1 AND 2 OR 3 OR 4 OR 5 OR 6 OR 7 OR 8	
SEARCH RESTRICTIONS	
Year	1970 – December 2020
Language	English
Source	Article
Author affiliation	New Zealand

### Data processing

**Fig. 1:**
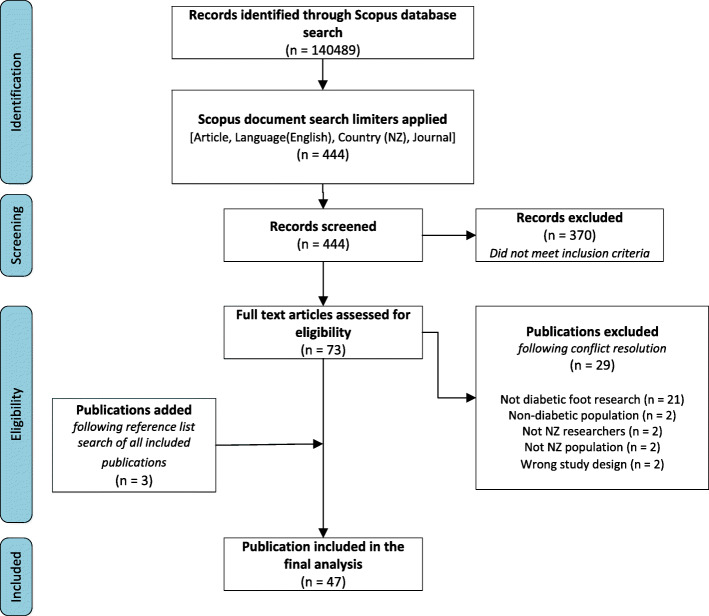
Flow chart for the search and retrieval process

The titles and abstracts of all identified publications were downloaded from Scopus® database and exported into the online systematic review application Rayyan (http://rayyan.qcri.org) [22]. The articles were then independently screened by two authors (MC, ISA) and selected based upon pre-determined inclusion and exclusion criteria agreed by all authors (Table 2). Any conflicts were discussed between two authors (MC and ISA) until consensus was achieved. A third author (KC) was available if conflicts were unable to be resolved, but this was not required. Additional publications were identified through backward snowballing of reference lists [23]. The study retrieval process is displayed in Fig. 1.

**Table 2:** Inclusion and exclusion criteria

**Inclusion criteria** Publications were included if: 1. They were original articles, or systematic reviews with meta-analysis; and 2. The research was conducted within an NZ institution; and 3. Data were reported that was conducted on an NZ population; and 4. The article had at least one author with an affiliation to an NZ research institution; and 5. They were published in English, and 6. The field of research was related to DRFD (including screening, prevention, diagnosis, management, complications, and workforce) and relevant conditions (peripheral neuropathy, neuroarthropathy, peripheral artery disease, infections, deformity, ulceration, and amputation); and 7. They were published between 1970 to the current date (date of search) **Exclusion criteria** The following studies were excluded: non-original research publications, non-systematic reviews, case reports, commentaries, letters, and editorials	

*DRFD* diabetes-related foot disease, *NZ* Aotearoa New Zealand

Common indicators of bibliometric analysis were extracted from each publication: title, year of publication, journal name, journal impact factor (IF), citation count, author names, total authors per manuscript, institutional affiliation, collaboration network and funding source [24]. Collaborative networks were classified into four categories: (1) “international collaborative” articles involving collaboration with international authors, (2) “bi-national link” articles originating from authors affiliated to only two NZ institutions, (3) “multi-national link” articles authored by researchers from three or more NZ institutions, and (4) “no collaboration” articles representing publications where all authors were affiliated to the same institution [21, 25]. Funding sources were classified into two categories: (1) industry sponsored funding, and (2) academic/public funding (funding derived from universities, hospitals, or government bodies). Using the 2019 International Working Group on the Diabetic Foot (IWGDF) guidelines as guidance, articles were classified into the following predetermined categories; (1) screening and prevention of DRFD, (2) management of DRFD related conditions, (3) epidemiology, and (4) other/miscellaneous (publications that did not fit into one of the above groups) [26]. Articles were also characterised by type of study (basic/clinical research articles, systematic review with meta-analyses, and randomised controlled trials (RCT)) [27].

As a measure of research quality, the journal IF attained in the year prior to publication was obtained using the Web of Science Journal Citation Reports™ tool (Clarivate Analytics, Philadelphia, Pennsylvania, USA). The number of citations per article was determined using the Scopus® database (Elsevier). All data were extracted into a custom Microsoft Excel spreadsheet, Version 2016 (Microsoft Corp., Redmond, Washington, USA) and also into the Biblioshiny software for additional analysis (based on R version 3.6.1, Bibliometrix package version 2.2.1; University of Naples Federico II, Naples, Italy, 2016) [28]. Biblioshiny was used to extract the following data: general characteristics of the included articles, annual scientific production, average citations per year, and most relevant authors, and collaboration world map. The nonparametric Mann-Kendall test was applied to data to detect statistically significant trends in publication numbers and collaborative networks. Statistical significance was determined as *p*-value (< 0.05).

## Results

The characteristics of the included studies are displayed in Table 3. A total of 140,489 publications were identified. Following application of the Scopus® search limiters [Article, Language (English), Country (NZ), Journal], 444 articles were assessed for inclusion (Fig. 1). After application of the study inclusion and exclusion criteria, 47 articles were included in the final analysis.

**Table 3:** Characteristics of the included studies (*n* = 47)

Variable	Number
Total number of articles	47
Average years from publication	9.62
Average citations per article	20.23
Average citations per year per article	2.56
References	1266
Total authors (range: 2–18)	300
Average co-authors per article	6.38
Unique authors (mean 4.81)	226
Single-authored articles	0

### Volume of scientific production

**Fig. 2:**
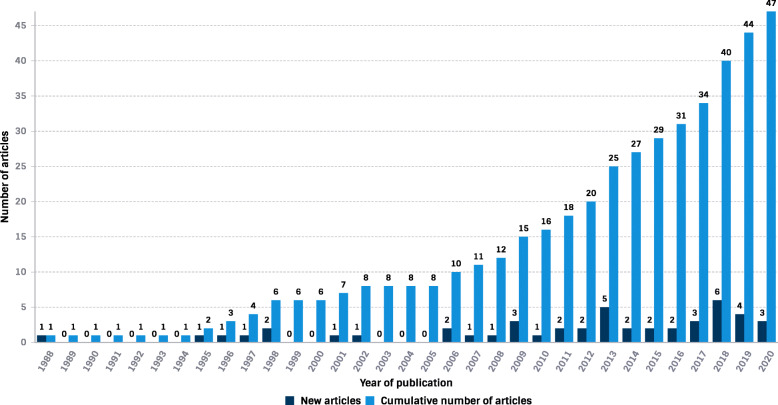
Graph showing the cumulative number and number of new articles of Aotearoa New Zealand diabetes-related foot disease-related publications per year between 1988 and 2020)

The volume of NZ-produced DRFD publications has steadily increased since 1988. The number of publications per year showed a statistically significant positive trend (τ_b_ = 0.66, *P* < 0.001) increasing from one in 1988 to a peak of 6 in 2018 (Fig. 2). The majority of articles (*n* = 31, 66%) were published in the last decade (2011–2020). The most articles published in one year were six in 2018, representing 13% of total publications. Of the 47 articles, the majority (87%) were focused on basic/clinical research, with six (13%) RCTs. No systematic reviews with meta-analysis were identified.

### Areas of research focus

When categorised by area of research, 14 (30%) investigated screening or prevention of DRFD, 15 (32%) management of diabetes-related foot complications, 16 (34%) the epidemiology of DRFD, and two studies (4%) investigated other aspects of DRFD (the financial burden of DRFD care in hospital settings and vasoconstrictive responses in the skin).

### Funding sources

Fifteen articles (32%) reported receiving funding support. Eleven articles (23%) reported receiving NZ based funding. Of these, 10 (21%) were funded through university/public sources, with one industry funded. Four (9%) international multi-centre studies reported funding from international sources.

### Author and authorship network

**Fig. 3:**
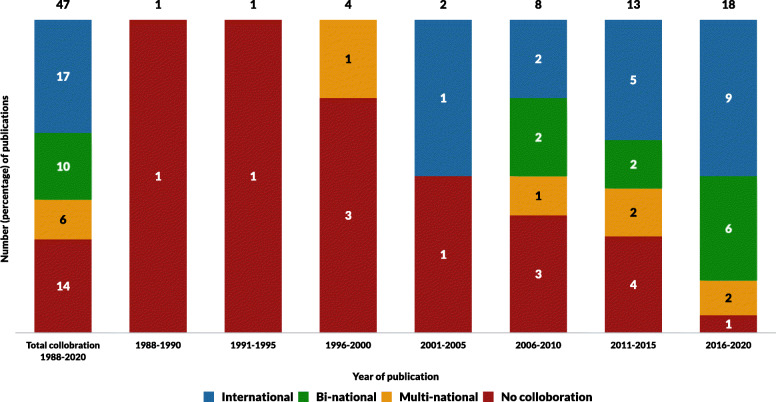
Graph showing authorship networks between 1988 and 2020

**Fig. 4:**
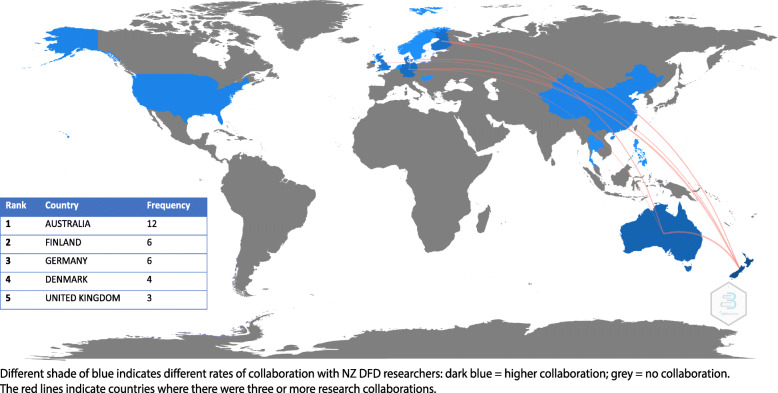
Visual representation of international collaborative authorship networks

Authorship networks for articles published between 1988 and 2020 are displayed in Fig. 3. No studies were single authored. Articles had a median of five co-authors per article (range: 2–18). Seventeen (36%) of the included articles had an international authorship link with a “bi-national link” in ten articles (21%), a “multi-national link” in six articles (13%), and “no collaborative link” outside of a single institution in 14 articles (30%). There was a significant increasing trend of international collaboration (τ_b_ = 0.47, *P* = 0.008) between 1988 and 2020, but no significant trend found in bi-national author collaboration (τ_b_ = − 0.035, *P* = 0.87) during the same time. Of the 39 articles published between 2006 and 2020, there were international links identified in 16 articles (41%) and bi-national links in 10 articles (26%). Of the 10 articles with bi-national links, eight (80%) were networks between universities and District Health Boards (DHBs). Eight articles were published between 1986 and 2005, six of which had no collaborative links. The most frequent international authorship links occurred between NZ and Australia (12 articles), Finland (6 articles) and Germany (6 articles). A world map displaying international collaborative research links by country is displayed in Fig. 4.

### Most common journals

The identified 47 articles were published in 27 different journals, of which the *New Zealand Medical Journal* (*n* = 10, 21%) and *Diabetes Research and Clinical Practice* (*n* = 4, 8%) were the most common. There were 18 journals (38%) where only one of these DRFD-related articles was published. The journals that published two or more publications accounted for 63% of all identified articles (Table 4).

**Table 4:** Frequency of publication of the most common journals that have published two or more Aotearoa New Zealand diabetes-related foot related research articles

Journal	n (%)
New Zealand Medical Journal	10 (21)
Diabetes Research and Clinical Practice	4 (9)
Diabetologia	3 (6)
Primary Care Diabetes	3 (6)
Diabetes Care	2 (4)
Gait and Posture	2 (4)
Journal of Foot and Ankle Research	2 (4)
Journal of Vascular Surgery	2 (4)

### Quality appraisal (impact factor and citations)

An IF was available for 34 (72%) of the journals where the included NZ based research articles were published. The median IF for the included studies was 4.31 (IQR: 2.75–6.81, range 1.82–79.32). The articles had a total of 951 citations, with an average citation of 20.2 per article (median: 10, IQR: 4–24, range: 0–209 citations). Three (6%) articles were cited once, and two articles (3%) had not been cited.

### Five most cited articles

The top five source journals are displayed in Table 5. The most cited article was published by Rajamani et al. [29] in *The Lancet* in 2009 (209 citations). This article represented 22% of the total citations for all included articles. The five most cited articles represented 46% of the total citations. Four of the most cited articles were international multi-centre RCTs. Only one of the five most cited studies had a NZ researcher as first author [33]. Except for Behrendt et al.’s observational study [31], all studies were supported by funding.

**Table 5:** Five most cited diabetes-related foot disease-related publications (1988–2020)

Author and Publication	First author country affiliation	Publication title	Journal	Number of citations	Total citations per year	Funding source
Rajamani et al. [29]	Australia	Effect of fenofibrate on amputation events in people with type 2 diabetes mellitus (FIELD study): a prespecified analysis of a randomised controlled trial	*The Lancet*	209	16.08	International
Herrman et al. [30]	Australia	Serum 25-Hydroxyvitamin D: A predictor of macrovascular and microvascular complications in patients with type 2 diabetes	*Diabetes Care*	86	12.28	International
Behrendt et al. [31]	Germany	International Variations in Amputation Practice: A VASCUNET Report	*European Journal of Vascular and Endovascular Surgery*	50	12.50	No funding declared
Chan et al. [32]	Australia	Plasma total bilirubin levels predict amputation events in type 2 diabetes mellitus: The Fenofibrate Intervention and Event Lowering in Diabetes (FIELD) study	*Diabetologia*	49	5.44	International
Dobson et al. [33]	NZ	Effectiveness of text message based, diabetes self-management support programme (SMS4BG): Two arm, parallel randomised controlled trial	*British Medical Journal*	46	11.50	NZ

Table 6 details the most cited articles where an author from NZ was the first author. With the exception of Dobson et al. [33], all were observational studies based in NZ [15, 33, 34]. Three studies declared funding from an academic/public source withing NZ while two studies were not supported financially [35, 36].

**Table 6:** Most cited diabetes-related foot disease-related publications with Aotearoa New Zealand researcher as a primary author cited between 1988 and 2020

Author and Publication	Publication title	Journal	Number of citations	Total citations per year	Funding
Dobson et al. [33]	Effectiveness of text message based, diabetes self-management support programme (SMS4BG): Two arm, parallel randomised controlled trial	*British Medical Journal*	46	11.50	Academic/public from NZ source
Nukada et al. [34]	Pathology of acute and chronic ischaemic neuropathy in atherosclerotic peripheral vascular disease	*The Brain*	44	1.69	Academic/public from NZ source
Bevan & Tomlinson [35]	Radiographic measures as a predictor of ulcer formation in diabetic Charcot midfoot	*Foot & Ankle International*	41	2.92	No funding declared
Misra et al. [36]	Peripheral neuropathy and tear film dysfunction in type 1 diabetes mellitus	*Journal of Diabetes Research*	40	5.0	No funding declared
Gurney et al. [15]	Risk of lower limb amputation in a national prevalent cohort of patients with diabetes	*Diabetologia*	29	7.25	Academic/public from NZ source

## Discussion

Bibliometric analyses are useful for inferring trends over time, themes researched, detection of the most prolific scholars and institutions and to present the “big picture” of research in a particular field [37]. The present study analysed the research quantity (i.e., publication output, areas of research focus and trends over time) and quality (i.e., the level of evidence, degree of collaboration, IF, and citation rates) of NZ DRFD-related research.

In line with an increasing worldwide volume of DRFD research [21] the number of NZ produced DRFD publications has steadily increased, albeit a relatively small increase, between 1988 and 2020. Notably, from 2006 onwards there was an increase in published articles with a peak of six new articles in 2018, and an increase in research growth and national collaboration. The underlying reasons for the increase in DRFD research are not attributable to any one factor but may have resulted from the increasing prevalence of diabetes in NZ [9] and the numerous diabetes quality of care strategies, policies and initiatives implemented in the early 2000s in NZ. Additional File 1 presents a timeline of policies/strategies/initiatives that may have potentially influenced NZ DRFD research. Notably during this period, the NZ Government strategy elevated the importance of diabetes with the release of The NZ Health Strategy (2001) [38]. Reducing the incidence and impact of diabetes was one of the 13 health objectives chosen for implementation in the short to medium term. Alternatively, the significant rise in the NZ-produced DRFD research may reflect the general worldwide trend of increasing scholarly activities across disciplines, including research focused on areas related to DRFD [39].

Whilst the significance of diabetes in NZ came to the fore with changes in health policy and strategy, research development prior to 2000 was hindered by limited research funding. In 2000, health research accounted for only 1% of the national health budget [40]. It was not until 2005 that the Health Research Council (HRC) of NZ became a Crown agent, charged with putting into effect government policy in relation to health research [41]. HRC now invests $NZ126 million a year into research studies, projects, and programmes [41]. The opportunities for NZ researchers to obtain funding have improved in the past 15 years, however, our results show DRFD research by NZ researchers is still poorly funded with only 32% of the included articles declaring research funding support. Most of these studies were funded by national organisations (DHBs, universities, HRC; *n* = 11, 23%) and represent studies that were largely observational. As these types of studies are of a lower level of evidence as characterised by the Oxford Centre for Evidence based Medicine levels of evidence [27], they are often considered insufficient to change clinical practice compared to RCTs that are considered to be the gold standard for demonstrating efficacy [42]. As shown by our analysis, there are few data related to DRFD disease derived from RCTs or interventional studies. Consequently, to bridge this gap, a medicine-based evidence approach may need to be adopted. The term medicine-based evidence, defined as a patient-centred approach to the evaluation of data that recognizes RCTs may not always yield higher-quality evidence than observational studies and/or provides high-quality evidence where RCT data are lacking [43]. In order for such an approach to be adopted, the development of comprehensive data registries and the generation of big data sets are required. This can only be achieved through further development of international research collaborations identified by this study and increased research funding from government organisations like the HRC of NZ. The impact of international research on NZ based provision of DRFD care must also be acknowledged. International research such as the Seattle Diabetic Foot Study [44], the North West diabetes foot care study [45], research examining multi-disciplinary-based diabetes foot ulcer care [46], and work from the Scottish Diabetes Foot Action Group in foot screening and risk stratification [47] has provided practice-changing evidence which is reflected in how diabetes foot care is provided in NZ.

The research collaboration post 2006 demonstrated marked growth with international and bi-national collaboration increasing. Bi-national authorship post 2006 may have been positively affected by increasing working relationships between DHBs, Primary Health Organisations (PHOs), and university research institutes, partially facilitated by health system restructures. Our data shows there has been a high level of bi-national collaboration since 2006, with 80% of national collaboration occurring between a DHB and university research institute/department. International collaborations indicate the increasing global reach of NZ based research and active exchange of knowledge and research skills.

Despite the increase in number of publications and increased national and international collaborations, the majority of the identified publications (87%) represent studies classified as basic/clinical research, which represents a lower level of evidence as per the Oxford Centre for Evidence-based Medicine (level 3 or 4 evidence) [48]. Based upon the Oxford levels of evidence rating, and the relatively low citations rates, the majority of identified NZ DRFD-related publications were categorised as of poor quality. Of the top five cited articles (contributing 46% of total citations), four were RCTs (one first-authored by an NZ researcher) and four were multi-centre studies first-authored and led by international researchers. The most cited articles were either multi-centre national or international RCTs representing high quality of evidence.

The median journal IF (4.31, IQR: 2.75–6.81) is reasonably high for included publications. A previous study by Al-Busaidi et al. investigating diabetic foot disease research in Gulf Cooperation Council countries that included 96 publications found a median IF of 0.15, compared to 4.31 from 47 NZ produced DRFD publications [21]. Analysis by SCI Journal in 2018 (https://www.scijournal.org/articles/good-impact-factor) found that only 2% of journals have an IF of 10 or more, and 13% with IF of 4 or more. An IF greater than 3.29 places a journal in the top 20% of medical and health profession journals. Only five articles were published in journals with an IF of greater than 10. Of note, most publications were published in the New Zealand Medical Journal (*n* = 10, 21%), which does not currently hold an IF. IF is the most common metric for evaluating bibliometric impact of published research, however the value of the research is not necessarily reflected by the IF [21]. This finding is interesting as researchers often seek publication in so called higher impact/prestigious journals intending to improve their personal citation rate, and h-indices. Furthermore, academic staff promotions at universities often depend upon the publication of a certain number of articles in scientific journals [49]. However, it is possible authors may choose to publish in journals based on the intended audience/readership where the article may have the most context and/or clinical impact. This may be more common when authors have a clinical rather than purely academic background, where their driver may be to improve clinical outcomes rather than produce high ranked research outputs. Alternatively, the decision to publish in a particular journal may be dictated by the availability of funding to support the fees associated with publication. A combination of these factors may be likely reasons for many NZ authored DRFD articles being published in the *New Zealand Medical Journal*.

Categorisation of research by type found that there were a relatively even spread number of articles categorised as screening/prevention, management of diabetes-related foot complications, and epidemiological studies. However, of the studies categorised as screening/prevention none were interventional studies. With few studies aimed at improved care or prevention of diabetes-related foot ulceration/amputation, coupled with recent international calls to reduce foot ulcer incidence by at least 75% and local NZ health priorities to reduce health inequities for Māori, a shift in DRFD research priorities is essential [6]. Therefore, the first steps towards this goal are to evaluate the performance of health services aimed at the prevention and early detection of DRFD, and the ability of services to reduce inequities in access to services and health outcomes. This is a priority in NZ given the regional variation in DRLEA and significantly higher amputation rates for Māori [7, 12, 13].

The results of this study have several limitations that must be considered. All metrics were extracted based upon our pre-defined search terms, and data only from the Scopus® database, which may not include all publications that meet our inclusion criteria. Some peer-reviewed journals are not indexed in Scopus®. However, we also checked for additional publications by screening reference lists of identified articles from the initial search. As this study included only journal articles, our findings may not reflect all NZ DRFD literature. It is acknowledged there may be grey literature sources that reveal a number of NZ based quality-of-care improvement initiatives related to DRFD that are not published in peer-reviewed journals. Finally, journal IF was used to assess the quality of published research, which has been debated as a research quality indicator [21]. However, IF is the most commonly used and arguably the best existing metric for evaluating the bibliometric impact of published research [50].

## Conclusion

DRFD articles authored by NZ researchers have increased over the past five decades. Despite NZ researchers increasing their global impact through collaborative networks, most of the research was classified as low-level evidence, with limited financial support and funding. Increased funding for interventional research is required to enable a higher level of NZ relevant, evidence-based, and practice-changing research to occur. Future research must focus on the NZ context and reducing inequalities in diabetes-related outcomes for Māori by specifically developing and evaluating interventions to better prevent, screen for, and manage DRFD in NZ.

## Supplementary Information

**Additional file 1:.** Significant policies/strategies/groups that have influenced New Zealand diabetes-related foot disease reasearch

## Data Availability

All data used in this article can be found on the Scopus database using the search strategy outlined in the Methods section. A complete list of all included papers in available upon reasonable request from the corresponding author.

## Abbreviations

NZ: Aotearoa New Zealand
DRFD: Diabetes-related foot disease
DHB: District Health Board
IF: Impact factor
DRLEA: Diabetes-related lower-extremity amputation
PHO: Primary health organisations
RCT: Randomised controlled trial
HRC: Health Research Council
