# Supplementary material for: Diabetes-related foot disease research in Aotearoa New Zealand: a bibliometric analysis (1970–2020)
Source: J Foot Ankle Res. 2022 Mar 21;15:23. doi: 10.1186/s13047-022-00528-5 (PMC8939115; doi:10.1186/s13047-022-00528-5)

Additional File 1 – Significant policies/strategies/groups that have influenced NZ DRFD reasearch

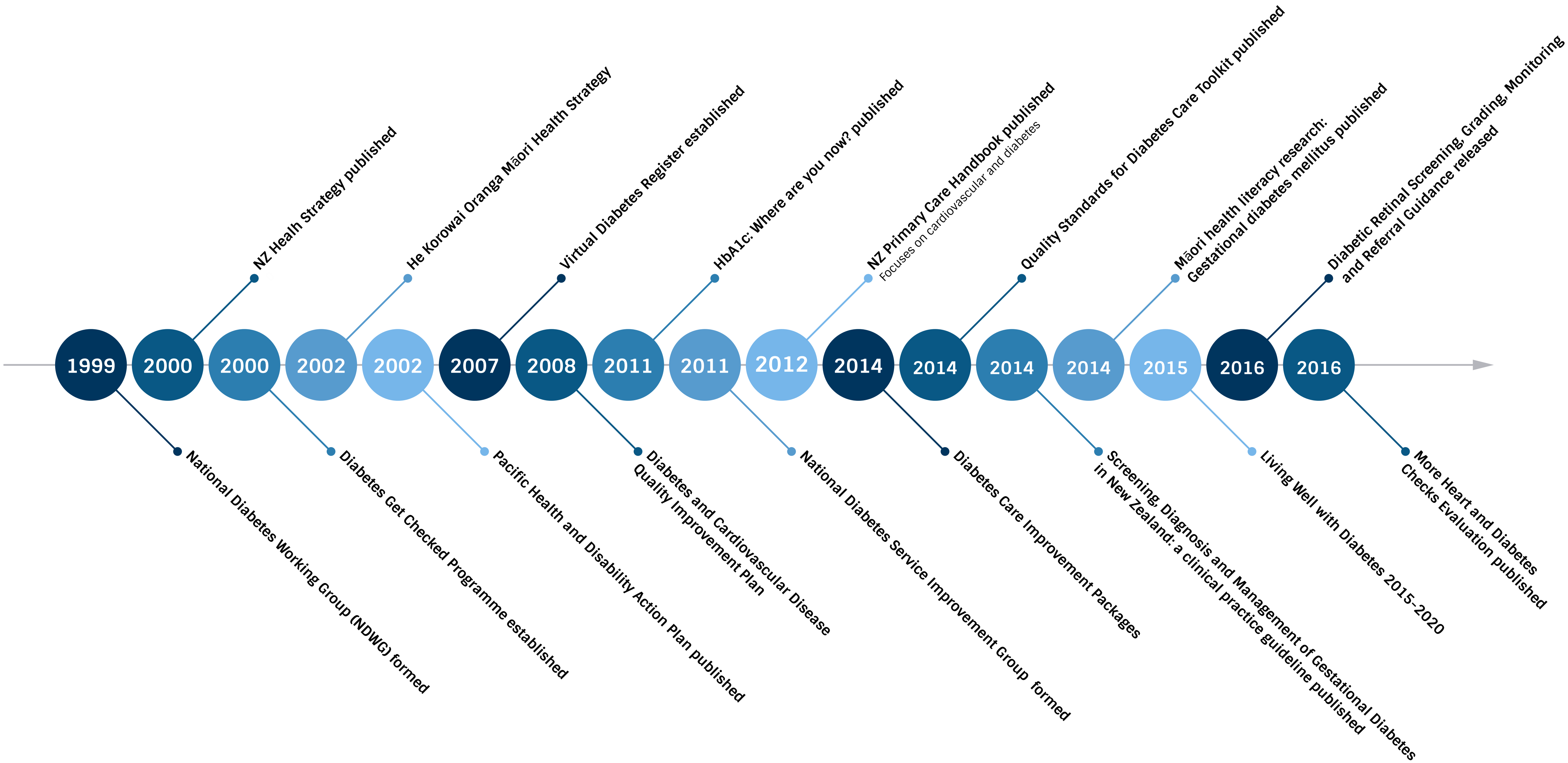

Supplement: Supplementary file 1 — Additional file 1:. Significant policies/strategies/groups that have influenced New Zealand diabetes-related foot disease reasearch [file 13047_2022_528_MOESM1_ESM.pdf]
